# PROPHYLACTIC AND LONG-LASTING THERAPEUTIC EFFICACY OF SENOLYTIC CAR T CELLS AGAINST AGE-RELATED PHENOTYPES

**DOI:** 10.1093/geroni/igae098.1287

**Published:** 2024-12-31

**Authors:** Corina Amor

**Affiliations:** Cold Spring Harbor Laboratory, Cold Spring Harbor, New York, United States

## Abstract

Senescent cells accumulate in organisms over their lifespan and play a key role in age-related tissue decline and the development of chronic aging pathologies. Thus, effective strategies to eliminate senescent cells (senolytics) could have broad therapeutic implications. In a departure from conventional chemical approaches we developed the first cell-based senolytic therapy based on chimeric antigen receptor (CAR) T cells targeting uPAR, a cell-surface protein upregulated on senescent cells. Our initial proof of concept showed their efficiency in young animal models of liver fibrosis and cancer. We now show that uPAR-positive senescent cells accumulate during physiological aging and characterize their cell types and expression profiles. Importantly, we find that they can be safely targeted with senolytic CAR T cells in aged animals where they result in significant improvements in both tissue regeneration and metabolic function. Of note, we find that the beneficial effects of senolytic CAR T cells are long lasting; single administration of a low dose is sufficient to safely achieve long-term therapeutic and preventive effects in healthspan.

